# Supplementary material for: Cave Pools in Carlsbad Caverns National Park Contain Diverse Bacteriophage Communities and Novel Viral Sequences
Source: Microb Ecol. 2024 Dec 26;87(1):163. doi: 10.1007/s00248-024-02479-9 (PMC11671562; doi:10.1007/s00248-024-02479-9)
Supplement: Supplementary file 1 — Supplementary file1 (DOCX 573 KB) [file 248_2024_2479_MOESM1_ESM.docx]

**Supplemental Information**

**Cave pools in Carlsbad Caverns National Park contain diverse bacteriophage communities and novel viral sequences**

***Microbial Ecology***

**Joseph Ulbrich**^1,2^ **· Nathaniel E. Jobe**^1^ **· Daniel S. Jones**^3,4^ (https://orcid.org/0000-0003-4556-0418) **· Thomas L. Kieft**^1^ (https://orcid.org/0000-0003-4350-9416)

Author for correspondence: Thomas L. Kieft

[thomas.kieft@nmt.edu](mailto:thomas.kieft@nmt.edu)

^1^Department of Biology, New Mexico Institute of Mining and Technology, Socorro, NM 87801 U.S.A.

^2^Current address: OpenEye Scientific, 9 Bisbee Court, Suite D, Santa Fe, NM 97508 U.S.A.

^3^Department of Earth and Environmental Science, New Mexico Institute of Mining and Technology, Socorro, NM 87801 U.S.A.

^4^National Cave and Karst Institute, Carlsbad, NM 88220, U.S.A

**Supplemental Table S1**  Elemental analysis of pool water by ICP-MS.

| Element  (mg L^-1^) | Longfellow’s Bathtub | Green Lake | Lower Cave Pool | Iron Pool |
| --- | --- | --- | --- | --- |
| Aluminum | BD^a^ | 0.0007 | BD^a^ | BD^a^ |
| Antimony | BD^a^ | BD^a^ | BD^a^ | BD^a^ |
| Arsenic | BD^a^ | 0.0010 | 0.0011 | BD |
| Barium | 0.089 | 0.259 | 0.311 | 0.090 |
| Beryllium | BD^a^ | BD^a^ | BD^a^ | BD^a^ |
| Boron | 0.073 | 0.061 | 0.047 | 0.492 |
| Cadmium | BD^a^ | BD^a^ | BD^a^ | BD^a^ |
| Calcium | ND^b^ | ND^b^ | 19.5 | 31.2 |
| Chromium | BD^a^ | BD^a^ | 0.0010 | BD^a^ |
| Cobalt | BD^a^ | BD^a^ | BD^a^ | BD^a^ |
| Copper | BD^a^ | 0.0026 | BD^a^ | BD^a^ |
| Iron | BD^a^ | BD^a^ | BD^a^ | BD^a^ |
| Lead | BD^a^ | BD^a^ | BD^a^ | BD^a^ |
| Lithium | 0.005 | 0.005 | 0.005 | 0.394 |
| Magnesium | BD^a^ | BD^a^ | 36.6 | 1850 |
| Manganese | BD^a^ | BD^a^ | BD^a^ | BD^a^ |
| Molybdenum | BD^a^ | 0.001 | 0.001 | 0.193 |
| Nickel | BD^a^ | 0.0012 | BD^a^ | BD^a^ |
| Potassium | ND^b^ | ND^b^ | 0.53 | 372 |
| Selenium | BD^a^ | 0.001 | 0.001 | 0.194 |
| Silicon | 18.9 | 8.99 | 9.29 | 1.21 |
| Silver | BD^a^ | BD^a^ | BD^a^ | BD^a^ |
| Strontium | 0.227 | 0.115 | 0.260 | 0.148 |
| Thallium | BD^a^ | BD^a^ | BD^a^ | BD^a^ |
| Thorium | BD^a^ | BD^a^ | BD^a^ | BD^a^ |
| Tin | BD^a^ | 0.0012 | 0.008 | BD^a^ |
| Titanium | BD^a^ | 0.001 | 0.001 | BD^a^ |
| Uranium | BD^a^ | 0.0013 | 0.0015 | BD^a^ |
| Vanadium | 0.0142 | 0.0096 | 0.0044 | BD^a^ |
| Zinc | 0.0213 | 0.0076 | 0.0128 | BD^a^ |

^a^BD, below detection limit

^b^ND, no data

**Supplemental Table S3 Diversity indices for prokaryotes based on amplicon sequencing data.**

| **Index** | **Lower Cave Pool** | **Longfellow’s Bathtub** | **Iron Pool** | **Green Lake** |
| --- | --- | --- | --- | --- |
| Shannon | 3.38 | 4.03 | 3.67 | 4.19 |
| Inverse Simpson’s (1/D) | 17.1 | 17.3 | 14.0 | 18.8 |
| Chao1 | 139 | 252 | 235 | 187 |

**Supplemental Table S4** List of potential cellular host species based on iPHoP analysis.

| **Contig** | **Length**  **(bp)** | **Host classification: domain (d), phylum (p), class (c), order (o), family (f), genus (g)** | **Confi-dence score** | **Method(s), with scores from individual method(s)** |
| --- | --- | --- | --- | --- |
| 86 | 54743 | d: *Bacteria*, d: *Desulfobacterota*, c: *Desulfobaccia*, o: *Desulfobaccales*, f: 0-14-0-80-60-11, g: 0-14-0-80-60-11 | 91.6 | BLAST, 93.90 |
| 161 | 46316 | d: *Bacteria*, d: *Acidobacteriota*, c: *Thermoanaerobaculia*, o: Gp7-AA8, f: Gp7-AA8, g: JADGNZ01 | 90.8 | BLAST, 93.20 |
| 167 | 46116 | d: *Bacteria*, d: *Bacillota*_A, c: *Clostridia*, o: *Oscillospirales*, f: *Ruminococcaceae*, g: *Ruminiclostridium*_E | 98.5 | CRISPR, 98.90 |
| 252 | 42485 | d: *Bacteria*, d: *Bacillota*_A, c: *Clostridia*, o: *Lachnospirales*, f: *Lachnospiraceae*, g: *Eubacterium*_G | 91.0 | iPHoP-RF, 93.40 |
| 356 | 39871 | d: *Bacteria*, d: *Bacillota*_A, c: *Clostridia*, o: *Lachnospirales*, f: *Lachnospiraceae*, g: *Blautia* | 90.3 | iPHoP-RF, 92.80 |
| 542 | 32336 | d: *Bacteria*, d: *Actinomycetota*, c: *Acidimicrobiia*, o: *Acidimicrobiales*, f: *Ilumatobacteraceae*, g: UBA2093 | 94.9 | BLAST, 96.40 iPHoP-RF, 62.50 |
| 608 | 29806 | d: *Bacteria*, d: *Pseudomonadota*, c: *Gammaproteobacteria*, o: *Pseudomonadales*, f: *Pseudomonadaceae*, g: *Pseudomonas*_E | 92.2 | iPHoP-RF, 94.40 |
| 898 | 20594 | d: *Bacteria*, d: *Pseudomonadota*, c: *Gammaproteobacteria*, o: *Burkholderiales*, f: *Burkholderiaceae*, g: *Paraburkholderia* | 93.5 | iPHoP-RF, 95.40 |
| 1250 | 14773 | d: *Bacteria*, d: *Bacillota*, c: *Bacilli*, o: *Lactobacillales*, f: *Carnobacteriaceae*, g: *Atopostipes* | 90.3 | iPHoP-RF, 92.80 |
| 1752 | 11111 | d: *Bacteria*, d: *Actinomycetota*, c: *Actinomycetia*, o: *Mycobacteriales*, f: *Mycobacteriaceae*, g: *Mycobacterium* | 92.6 | iPHoP-RF, 94.70 |
| 1873 | 10561 | d: *Bacteria*, d: *Bacillota*, c: *Bacilli*, o: *Bacillales*, f: DSM-18226, g: *Neobacillus* | 91.0 | iPHoP-RF, 93.40 |
| 2975 | 6897 | d: *Bacteria*, d: *Actinomycetota*, c: *Actinomycetia*, o: *Mycobacteriales*, f: *Mycobacteriaceae*, g: *Mycobacterium* | 93.9 | iPHoP-RF, 95.70 |
| 3293 | 6352 | d: *Bacteria*, d: *Bacillota*_A, c: *Clostridia*, o: *Oscillospirales*, f: *Acutalibacteraceae*, g: *Ruminococcus*_E | 98.1 | CRISPR, 98.60 iPHoP-RF, 53.60 |
| 4088 | 5251 | d: *Bacteria*, d: *Actinomycetota*, c: *Actinomycetia*, o: *Propionibacteriales*, f: *Propionibacteriaceae*, g: WQYJ01 | 90.3 | iPHoP-RF, 92.80 |
| 4177 | 5166 | d: *Bacteria*, d: *Pseudomonadota*, c: *Gammaproteobacteria*, o: *Burkholderiales*, f: *Burkholderiaceae*_B, g: *Polaromonas* | 93.9 | iPHoP-RF, 95.70 |
| 4555 | 4811 | d: *Bacteria*, d: *Pseudomonadota*, c: *Gammaproteobacteria*, o: *Enterobacterales*_A, f: *Shewanellaceae*, g: *Shewanella* | 91.8 | iPHoP-RF, 94.10 |
| 5026 | 4417 | d: *Bacteria*, d: *Bacteroidota*, c: *Bacteroidia*, o: *Flavobacteriales*, f: *Flavobacteriaceae*, g: *Winogradskyella* | 90.3 | iPHoP-RF, 92.80 |
| 5066 | 4389 | d: *Bacteria*, d: *Bacteroidota*, c: *Ignavibacteria*, o: *Ignavibacteriales*, f: *Melioribacteraceae*, g: DSXH01 | 90.9 | BLAST, 93.30 iPHoP-RF, 51.50 |
| 5541 | 4044 | d: *Bacteria*, d: *Pseudomonadota*, c: *Alphaproteobacteria*, o: *Rhizobiales*, f: *Xanthobacteraceae*, g: *Bradyrhizobium* | 95.3 | iPHoP-RF, 96.70 |
| 5885 | 3846 | d: *Bacteria*, d: *Actinomycetota*, c: *Actinomycetia*, o: *Mycobacteriales*, f: *Mycobacteriaceae*, g: *Corynebacterium* | 90.3 | iPHoP-RF, 92.80 |
| 5931 | 3820 | d: *Bacteria*, d: *Pseudomonadota*, c: *Gammaproteobacteria*, o: *Pseudomonadales*, f: *Moraxellaceae*, g: *Psychrobacter* | 90.7 | iPHoP-RF, 93.10 |
| 6086 | 3742 | d: *Bacteria*, d*: Chloroflexota*, c: *Anaerolineae*, o: *Aggregatilineales*, f: *Phototrophicaceae*, g: OLB13 | 92.2 | iPHoP-RF, 94.40 |
| 6094 | 3736 | d: *Bacteria*, d: *Patescibacteria*, c: *Paceibacteria*, o: UBA9983_A, f: UBA1539_A, g: UBA1550 | 93.1 | iPHoP-RF, 95.10 |
| 6094 | 3736 | d: *Bacteria*, d: *Patescibacteria*, c: *Paceibacteria*, o: UBA9983_A, f: SBAW01, g: SBAW01 | 91.8 | iPHoP-RF, 94.10 |
| 6772 | 3447 | d: *Bacteria*, d: *Actinomycetota*, c: *Actinomycetia*, o: *Mycobacteriales*, f: Mycobacteriaceae, g: *Corynebacterium* | 95.3 | iPHoP-RF, 96.70 |
| 6997 | 3359 | d: *Archaea*, d: *Thermoproteota*, c: *Bathyarchaeia*, o: B26-1, f: BA1, g: BA2 | 93.5 | iPHoP-RF, 95.40 |
| 6997 | 3359 | d: *Archaea*, d: *Thermoproteota*, c: *Bathyarchaeia*, o: B26-1, f: BA1, g: BA1 | 92.6 | iPHoP-RF, 94.70 |
| 6997 | 3359 | d: *Archaea*, d: *Thermoproteota*, c: *Bathyarchaeia*, o: B26-1, f: BA1, g: Kmv02 | 90.7 | iPHoP-RF, 93.10 |
| 9110 | 2721 | d: *Bacteria*, d: *Chloroflexota*, c: *Dehalococcoidia*, o: UBA6952, f: QGNO01, g: QGNO01 | 93.5 | iPHoP-RF, 95.40 |
| 10653 | 2412 | d: *Bacteria*, d: *Actinomycetota*, c: *Actinomycetia*, o: *Actinomycetales*, f: *Actinomycetaceae*, g: *Pauljensenia* | 92.6 | iPHoP-RF, 94.70 |
| 10955 | 2356 | d: *Bacteria*, d: *Pseudomonadota*, c: *Gammaproteobacteria*, o: *Xanthomonadales*, f: *Rhodanobacteraceae*, g: *Luteibacter* | 91.8 | iPHoP-RF, 94.10 |
| 11146 | 2325 | d: *Bacteria*, d: *Actinomycetota*, c: *Actinomycetia*, o: *Actinomycetales*, f: *Micrococcaceae*, g: *Paenarthrobacter* | 94.5 | iPHoP-RF, 96.10 |
| 11945 | 2199 | d: *Bacteria*, d: *Bacteroidota*, c: *Bacteroidia*, o: *Bacteroidales*, f: *Muribaculaceae*, g: JAGBWK01 | 90.3 | iPHoP-RF, 92.80 |
| 12142 | 2173 | d: *Bacteria*, d: *Bacteroidota*, c: *Bacteroidia*, o: *Bacteroidales*, f: *Bacteroidaceae*, g: *Prevotella* | 92.6 | iPHoP-RF, 94.70 |
| 12324 | 2148 | d: *Bacteria*, d: *Pseudomonadota*, c: *Gammaproteobacteria*, o: *Enterobacterales*, f: *Enterobacteriaceae*, g: *Serratia* | 92.6 | iPHoP-RF, 94.70 |
| 12706 | 2102 | d: *Bacteria*, d: *Actinomycetota*, c: *Actinomycetia*, o: *Mycobacteriales*, f: *Mycobacteriaceae*, g: *Nocardia* | 95.3 | iPHoP-RF, 96.70 |


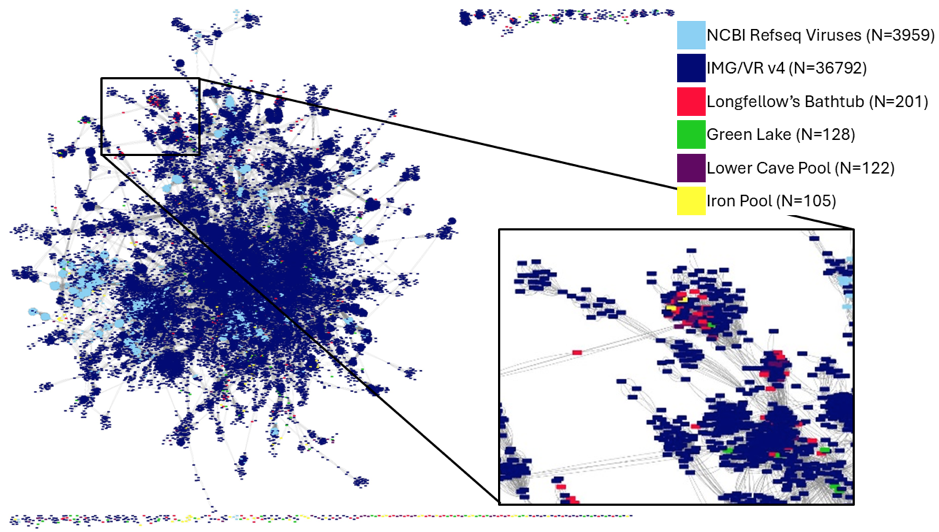


**Supplemental Fig. S1.** Gene-sharing network generated using vConTACT2 amended with viruses from IMG/VR. Colors are the same as in Fig 4 with the addition of dark blue for the viruses from IMG/VR. Subsection of cluster map enlarged to show clustering that is hard to see in the full image. Counts represent the number of nodes from each sample present on the cluster map.


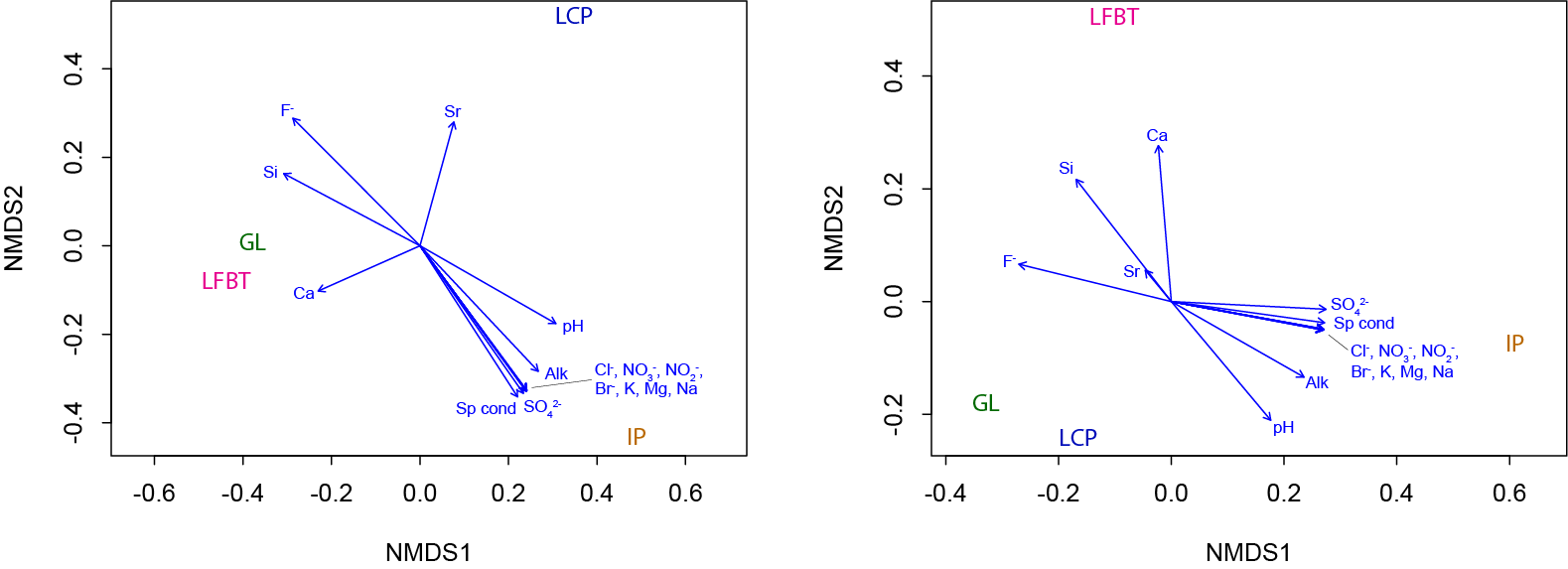


**Supplementary Fig S2** Nonmetric multidimensional scaling ordinations of virus communities (left) and microbial communities (right). Stress = 0 for both ordinations. Vectors show an overlay of environmental variables. Alk = alkalinity, sp cond = specific conductivity. The vectors for chloride, nitrate, nitrite, bromide, potassium, magnesium, and sodium overlap in both ordinations.
